# Emblem Gestures Improve Perception and Evaluation of Non-native Speech

**DOI:** 10.3389/fpsyg.2020.574418

**Published:** 2020-09-22

**Authors:** Kiana Billot-Vasquez, Zhongwen Lian, Yukari Hirata, Spencer D. Kelly

**Affiliations:** ^1^Department of Psychological and Brain Sciences, Colgate University, Hamilton, NY, United States; ^2^Center for Language and Brain, Hamilton, NY, United States; ^3^Linguistics Program, Colgate University, Hamilton, NY, United States; ^4^Department of East Asian Languages, Colgate University, Hamilton, NY, United States

**Keywords:** speech processing, non-native accent, hand gesture, multimodal, second language, cross-cultural communication

## Abstract

Traditionally, much of the attention on the communicative effects of non-native accent has focused on the accent *itself* rather than how it functions within a more natural context. The present study explores how the bodily context of co-speech emblematic gestures affects perceptual and social evaluation of non-native accent. In two experiments in two different languages, Mandarin and Japanese, we filmed learners performing a short utterance in three different within-subjects conditions: speech alone, culturally familiar gesture, and culturally unfamiliar gesture. Native Mandarin participants watched videos of foreign-accented Mandarin speakers (Experiment 1), and native Japanese participants watched videos of foreign-accented Japanese speakers (Experiment 2). Following each video, native language participants were asked a set of questions targeting speech perception and social impressions of the learners. Results from both experiments demonstrate that familiar—and occasionally unfamiliar—emblems facilitated speech perception and enhanced social evaluations compared to the speech alone baseline. The variability in our findings suggests that gesture may serve varied functions in the perception and evaluation of non-native accent.

## Introduction

More than half of the world’s population is bilingual, a pattern that has only accelerated since the turn of the millennium (Grosjean, 2010). Studies focused on the treatment and perception of non-native accented speech have shown that it is consistently discriminated against, negatively affecting measures related to likeability, sociability, and intelligence (Bradac, 1990; Lindemann, 2003). In an effort to understand accented speech within a natural communicative context, the present study explores how non-native accents are perceived and evaluated in the presence of co-speech emblematic gestures. Building on research demonstrating that gesture’s semantic relationship with speech can powerfully affect language processing, comprehension and learning (Church et al., 2017), the present study asks how a gesture’s *cultural* relationship to speech influences cross-cultural perceptions and impressions of accented speech and speakers.

### The Stigma of Accent

Many people learn their non-native language later in life—through formal education or pressures from commerce—so it is commonplace to speak a second language with a non-native accent (Johnson and Newport, 1989; Cheng, 1999). In general, a non-native accent, a term interchangeable with foreign accent, has been defined as “speech that systematically diverges from native speech due to interference from the phonological and acoustic-phonetic characteristics of a talker’s native language” (Atagi and Bent, 2017).

Unfortunately, non-native accents often carry a social stigma (Gluszek and Dovidio, 2010). Because accents are one of the most immediate, powerful and fixed cues to one’s cultural identity (Giles, 1977), they can reinforce and maintain stereotypes and prejudices between groups of people (Kinzler et al., 2007). In addition, they can be used as salient markers of socio-economic class and educational levels, which can lead native speakers to have a sense of superiority or inferiority compared to non-native accented speakers (Lippi-Green, 2012). Lippi-Green points out that this social hierarchy is so powerful that even the medical community treats the elimination of accents as an explicit goal in certain practices of speech therapy. Because native speakers and non-native speakers interact with one another more than ever (Cheng, 1999; Pickering, 2006), this leads to important questions about how this stigma plays out in social interactions and judgments within cross-cultural contexts.

Research investigating the perceptions and impressions of non-native accented speech has repeatedly shown that it is perceived less favorably than native accented speech on measures of believability (Lev-Ari and Keysar, 2010) and social preference (Kinzler et al., 2009, 2011; DeJesus et al., 2017). For example, Lev-Ari and Keysar (2010) found that people judged statements delivered by non-native accented speakers as less believable than when delivered by native accented speakers. In another study, social preference was measured by asking 5-year-old children to evaluate the likelihood of becoming friends with other children (Kinzler et al., 2009). The study found that, while American children chose the pictures of children with the same race when they were presented silently, they chose the pictures of children with the different race over those with the same race when the latter was speaking in French-accented English. Moreover, in a study that controlled for comprehensibility of non-native accents by using nonsense speech, researchers found that preschool-aged children sought and endorsed information from native accented speakers over non-native accented speakers (Kinzler et al., 2011). Because they used nonsense speech, this study revealed that comprehensibility was not a factor in the children’s choices; rather, the preference was driven solely by the sound of the speech itself. Together, these studies show that speaking with a non-native accent comes at a significant social cost.

### Hand Gestures and Native Language (L1)

Research has largely focused on how native and non-native accents interact with other cues to identity, like the race of the speaker (e.g., Rubin, 1992; Kinzler et al., 2011; DeJesus et al., 2017; Hansen et al., 2017). However, there is room for more research in the fluid aspects of communication that accompany accented speech, such as bodies, hands, and facial expressions that are a ubiquitous context when people speak (Kendon, 2004). For example, co-speech hand gesture—the natural movements of the hands and arms to co-construct meaning—is an essential component of everyday communication, so much so that some have theorized it should be treated as an integral part of language itself (McNeill, 1985, 1992, 2006). This fusion between speech and gesture justifies the importance of researching the two *together* when investigating all aspects of speech communication.

The integrated relationship between speech and gesture in languageproduction has led many researchers to study how these two parts ofthe system work together during language comprehension (for reviews, see Hostetter, 2011; Kelly, 2017). Specifically testing McNeill’s theory, Kelly et al. (2010) advanced the integrated systems hypothesis to show that that the semantic relationship between speech and gesture affect the accuracy and speed of language comprehension. Moreover, this semantic contribution appears to be bi-directional—gesture not only clarifies the meaning of speech, but speech itself clarifies the meaning of gesture. This tight relationship between speech and gesture has been further bolstered by research showing that speech and gesture are semantically integrated in traditional language networks in the brain (Willems et al., 2007; Wu and Coulson, 2007; Dick et al., 2009; Green et al., 2009; Holle et al., 2010).

Beyond semantics, co-speech gesture also serves a lower-level perceptual function as well. Indeed, researchers have shown that hand movements play a role in motor and acoustic processes, such as vocal production (Pouw et al., 2020) and prosodic accentuation (Krahmer and Swerts, 2007). For example, Krahmer and Swerts (2007) found that producing beat gestures with speech not only enhances acoustic properties of speech production, but they also help listeners perceive words to be more acoustically prominent in sentences, even when only the audio is presented. Moreover, when viewing beats, these gestures serve to enhance how viewers perceive prosodic stress in speech. On the neural level, this perceptual focusing function of gesture is evident in neuroimaging research showing that there tight coupling of gesture and speech during early stages of speech processing (Dick et al., 2009; Hubbard et al., 2009; Biau and Soto-Faraco, 2013; Wang and Chu, 2013; Skipper, 2014). In one early study, Hubbard et al. (2009) investigated the relationship between gesture and speech in the auditory cortex and found that compared to “speech with a still body” and “speech with nonsense hand gesture,” speech accompanied by a congruent gesture elicited greater activation of auditory areas in the brain, such as the left hemisphere primary auditory cortex and the planum temporale (see also Dick et al., 2009).

This tight connection between viewing the hands and perceiving speech make gestures a useful tool in “speechreading,” the ability to use visual cues of speakers to clarify what they are saying. In a pioneering (and under-cited) study, Popelka and Berger (1971) investigated how phrases presented in varying gesture conditions—ranging from no gesture to semantically congruent and incongruent iconic and deictic gestures—affected accurate perception of spoken sentences. They found that sentences presented with congruent gestures produced higher accuracy for hearing a spoken sentence than did sentences presented with no gestures, and both produced better accuracy than sentences accompanied by incongruent gestures. More recently, Drijvers and Özyürek (2017) discovered that when the auditory information is degraded, listeners particularly benefit from iconic gestures during speech comprehension (for similar evidence with people who are hard of hearing, Obermeier et al., 2011, or with “cued speech” representing the individual sounds of words with hands, LaSasso et al., 2003). However, when auditory information is too degraded, the “additive effect” from hand gestures is lost. So, it appears that not only do co-speech gestures help with understanding the meaning of an utterance, they also facilitate lower levels perceptual identification of the speech stream itself.

### Hand Gestures and Second Language (L2)

Hand gestures are just as much part of using an L2 as they are using an L1 (Neu, 1990; Gullberg, 2006; McCafferty and Stam, 2009). Indeed, Gullberg argues that, given the integrated relationship between speech and co-speech gestures, the latter should be viewed as a fundamental part of the L2 elements that learners must master when acquiring an L2. Just as there are proper ways to phonetically articulate L2 syllables and syntactically organize L2 sentences, there seem to be fitting ways to move the hands when speaking a different language (Kita, 2009; Özyürek, 2017). This appropriate use of gesture applies to more than just the nuts and bolts of L2 phonetics, vocabulary and grammar—it also has pragmatic and cultural functions. In Gullberg’s own words, “[t]he command of the gestural repertoire of a language is important to the individual learners’ communicative efficiency and ‘cultural fluency’ (Poyatos, 1983)—perhaps less in terms of misunderstandings (Schneller, 1988) than in terms of the general integration in the target culture” (Gullberg, 2006, p. 116).

Many of the experiments on this topic have focused on how L2 learners attend to information conveyed through the hands when perceiving novel speech sounds (Hannah et al., 2017; Kelly, 2017; Kushch et al., 2018; Baills et al., 2019; Hoetjes et al., 2019) and comprehending new vocabulary (Allen, 1995; Sueyoshi and Hardison, 2005; Sime, 2006; Kelly et al., 2009; Morett, 2014; Morett and Chang, 2015; Baills et al., 2019; Huang et al., 2019). For example, Kelly et al. (2009) investigated how semantic congruence of gesture and speech affected the learning of L2 Japanese vocabulary in native English speakers. Results from a free recall and recognition test showed that compared to speech alone, congruent gestures enhanced memory and incongruent gesture disrupted it (and see Hannah et al., 2017, for a similar effect in L2 phonetic processing). Based on research in this vein, Macedonia (2014) makes a strong case for why hand gestures should be a bigger part of the L2 classroom and language education more generally.

But what about the other side of the coin? How do gestures produced by L2 speakers *themselves* affect native speaker’s perceptions and impressions of those L2 speakers? There are a few notable studies that have addressed this question (Neu, 1990; Gullberg, 1998; Jungheim, 2001; Gregersen, 2005; McCafferty and Stam, 2009). For example, Gullberg (1998) observed that the more L2 learners produced co-speech gestures—particularly, iconic gestures—the more native speakers judged them to be generally proficient in the L2. This fits well with L1 research showing that co-speech gestures positively influence social evaluations of native speakers (Maricchiolo et al., 2009). And there is even some recent evidence that training L2 speakers to use co-speech gesture not only enhances impressions of those speakers, but also how those speakers actually produce L2 speech (Gluhareva and Prieto, 2017; Zheng et al., 2018; Hoetjes et al., 2019). For example, Gluhareva and Prieto showed that when native Catalan speakers were given training on how to pronounce English words with beat gestures, their L2 speech was judged by native English speakers to have improved significantly compared to when there was no training with beat gestures. Note that native speakers’ judgments were on L2 speech alone, where they did not see learners’ gestures. Thus, it remains to be seen if *viewing* L2 gestures affects how native speakers process lower level auditory aspects of L2 speech, such as, correctly hearing what was said or explicitly evaluating the non-native accent itself. In other words, it is possible that seeing L2 gestures not only helps to boost native speakers’ social impressions of an L2 learner, it may also help them make better sense out of what they are hearing.

### The Present Study

The present study explores this issue by focusing on a type of gesture that plays a powerful role in cross-cultural communication: emblematic gestures. Emblems are conventionalized movements of the hands, head and body that are understood by most members of one culture (or subculture), but not necessarily another (Efron, 1941; Ekman, 1972; Kendon, 1997; Kita, 2009; Matsumoto and Hwang, 2013). For example, in Japan, the emblem for, ‘It’s spicy,” is to hold the bridge of the nose with the thumb and index finger. Without culinary knowledge that wasabi causes a (strangely satisfying) burning sensation in the sinuses, this gesture would be quite baffling.

Emblems are interesting in an L2 context for a number of reasons. For one, they can be used simultaneously with L2 speech to create multimodal signals, and this allows L2 speakers to display additional knowledge about the L2 culture (Neu, 1990; Jungheim, 2001; Gullberg, 2006; Matsumoto and Hwang, 2013). Second, even though emblems are similar to words in that both have highly conventionalized forms, most emblems are less arbitrary than spoken words and exhibit an element of iconicity that more directly maps onto their cultural meaning (as with the “spicy” example) (McNeill, 1992; Poggi, 2008).^1^ This gives L2 speakers an additional opportunity to convey meaning (similar to co-speech iconic gestures), which is particularly useful if their pronunciation is below the native level. And third, compared to the phonological challenges of L2 speech, emblems are relatively simple and easy to learn, making these visual conventions very handy in cross-cultural communication (Matsumoto and Hwang, 2013).

Emblems have not received much attention in the study of L1 speech comprehension, likely because they often occur independently of speech (Goldin-Meadow, 1999). However, in an L2 context, speakers can intentionally use culture-specific emblems along with speech to supplement the meaning of their utterances, in addition to demonstrating their sensitivity and knowledge of the L2 culture. Because viewing co-speech emblems helps L2 speakers comprehend L2 utterances (Allen, 1995), it is likely that they also help L1 speakers understand the non-native speech of L2 learners.

Building on this previous work, we ask the following question: From the perspective of native speakers, how does the cultural familiarity of L2 emblems affect phonetic perception of non-native accented speech specifically, in addition to the more general social evaluation of non-native speakers? This work extends the literature in three ways. First, previous studies on the perceptual processing and social stigma of accent (e.g., Gluszek and Dovidio, 2010; Lev-Ari and Keysar, 2010; Lippi-Green, 2012) have largely excluded its natural multimodal communicative context. If appropriately using hand gestures is an integral part of learning a complete L2 repertoire, as Gullberg (2006) argues, it makes sense to expand the focus and study non-native accents in their fully embodied form. Second, because many emblematic gestures are based on distinct and learned conventions—which often vary by culture—it is possible to explore the consequences of L2 speakers producing culturally right or wrong emblems. Just as a gesture’s iconic meaning matters for L2 vocabulary learning (Kelly et al., 2009), it is possible a gesture’s *cultural* meaning matters for perceptions and evaluations of L2 speech. Third, although previous research has shown that producing co-speech gestures in an L2 can make a general positive impression on native speakers—for example, Gullberg (1998) showing that gestures make L2 speakers appear more proficient—no study to our knowledge has more specifically broken down how L2 hand gestures influence the processing of non-native accents *per se* separately from the influence of gesture on evaluations of learners themselves.

In two experiments in two different languages, Mandarin and Japanese, we investigate how different gesture-speech relationships affect the evaluation of foreign language accent and learner from the perspective of native speakers. Specifically, we created gesture-speech pairs in which emblems that accompanied L2 speech were either culturally *familiar* or *unfamiliar* to native Mandarin or Japanese speakers. For both experiments, L2 learners were filmed performing a short utterance in three different conditions: culturally familiar gesture (common in China or Japan), culturally unfamiliar gesture (uncommon in China or Japan), and speech alone. In a within-subjects design, native Mandarin speakers watched videos (across all conditions) of L2 Mandarin learners, and native Japanese participants watched videos of L2 Japanese learners. Following each video, participants were asked a set of questions targeting speech perception and social impressions of the L2 learners.

We made two predictions about how L2 learners’ gesture would affect L1 listeners’ perception of speech and social impressions.

(1) We predicted that, relative to speech alone, culturally familiar gestures would improve accuracy and foreign accent ratings of L2 speech, and would positively affect social impressions of the accented speaker.

(2) In contrast, culturally unfamiliar gestures, relative to speech alone, would decrease accuracy and foreign accent ratings of L2 speech, and would negatively affect social impressions of the accented speaker.

These predictions were based on the following two lines of research as summarized in the introduction: one line of research showing that the relationship of gestures to speech matters for phonetic and semantic comprehension in L1 (Popelka and Berger, 1971; McNeill et al., 1994; Kelly et al., 2010) and L2 (Kelly et al., 2009; Hannah et al., 2017), and another line of research showing that the presence of meaningful gestures helps manage social impressions of L1 (Maricchiolo et al., 2009) and L2 speakers (Gullberg, 1998; Gregersen, 2005; McCafferty and Stam, 2009).

## Experiment 1: Mandarin

### Methods

#### Participants

Thirty-six undergraduates (13 males and 23 females) from a small liberal arts university on the East Coast participated in Experiment 1. All participants were international students from different regions of mainland China. They were all judged by one of the authors from Beijing to be native Mandarin Chinese speakers. All of them learned English in school in China, and none grew up speaking it at home. None of their first exposure to English in the U.S. is earlier than age 15, but they scored 100 or higher in Test of English as a Foreign Language (TOEFL) at the time of admission to college. Participants received either academic credit in psychology or $5 in cash for their participation.

#### Materials

##### L2 learner stimuli

For the L2 learner video stimuli, we recruited twenty-one (14 males and 7 females) “learners” of Mandarin, who were students attending a small liberal arts university on the U.S. East Coast. None of the learners were native Mandarin speakers, and included a range of speaking Mandarin for the first time to those in intermediate and advanced level courses. The range of L2 Mandarin competency was intended to reflect varying levels of the Mandarin accent. Additionally, stimulus learners represented a wide range of racial, ethnic and gender diversity.

##### Video clips

**FIGURE 1:**
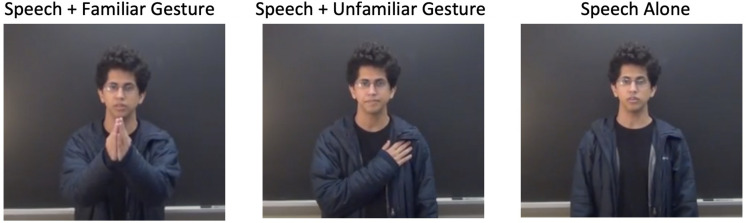
Stimuli example from Experiment 1: Sorry (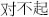 duì bu qǐ).

The stimuli in the experiment consisted of twenty-one 2–4 s videos of Mandarin phrases that are common in everyday speech (see Appendix 1 Supplementary Materials). Each phrase was produced by a different learner in three conditions: (a) Speech + Culturally Familiar Gesture, (b) Speech + Culturally Unfamiliar Gesture, and (c) Speech Alone. The “culturally familiar” gesture was defined as emblems that were familiar and commonly understood within Northern Mainland China. For example, consider the Mandarin utterance, “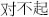 duì buqĭ,” which means “Sorry.” The culturally familiar emblem that goes with that speech is both palms meeting below the chin of the speaker, as in the left panel of Figure 1. A list of culturally familiar gestures was created with the help of native Mandarin speakers and Gestpedia^2^, a website that documents gestures from various locations and cultures. To generate “culturally unfamiliar” gestures, we also consulted Gestpedia to find emblems for our various phrases that were associated with various different cultures. Some of these were taken from American culture, but some other cultures include Japanese, Nigerian, Vietnamese, and Egyptian. For example, a culturally unfamiliar gesture to native Mandarin speakers was a palm touching the chest, as in the middle panel of Figure 1. After an extensive list was compiled, the gestures were screened by three separate native Mandarin speakers to assure cultural familiarity.

During the recording phase, one of the authors, whose L1 is Mandarin Chinese, was present to ensure that learners’ pronunciation of their assigned phrases were correct enough as to not to accidentally say a different word or phrase. Each learner said only one phrase but repeated it in the three conditions—familiar gesture, unfamiliar gesture, and speech alone—and all were videotaped. The stimulus clips were edited in Final Cut Pro and background noise in the audio clips was reduced with Audacity. In addition, the video clips were edited to have the same speech across all three conditions. To do this, the audio from the speech alone condition was dubbed onto all the other two versions of a given video to equate the speech across all conditions. This was important because it is known that producing hand gestures affects vocal production (Krahmer and Swerts, 2007; Pouw et al., 2020). Equally important was the naturalness of the audio and visual coupling. For this, we tested three people who were naïve to this experiment, and found that the stimuli all looked natural, and none of them noticed the dubbing. In summary, we created a total of 63 video clips (21 speakers × 3 conditions).

To prepare for the actual presentations of these video clips, three versions were created (see Appendix 2 Supplementary Materials), with the intention that each native speaker participant would take only one version of the experiment. Version A, B, or C each included all of the 21 learners, which meant that each version included all of the 21 utterances. But within each version, a learner appeared only in one of the three conditions. The condition in which the learner appeared was counterbalanced across the three versions. This was necessary to ensure that utterance type and gesture condition were not confounded, which is particularly important because there was a large range of accents across learners. In this way, we can control for diversity of accents by having each learner serve as his or her own control.

#### Evaluation of Learners’ Videos

A set of eight questions was used in the questionnaire. They were grouped into two general categories of evaluation: (1) questions that measured perception of speech itself (*speech* evaluation) and (2) questions about social impressions of the Mandarin learners (*learner* evaluation).

##### Speech evaluation

To measure various forms of speech perception, the following questions were presented in Mandarin Chinese, which was the participants’ L1: (1) *Words Misheard:* “What did this person say?” (fill in the blank); (2) *Accent:* “How would you rate their accent?” (1 = completely foreign to 10 = completely native Mandarin); and (3) *Tone Accuracy:* “How would you rate their tonal pronunciation?” (1 = completely incorrect to 10 = completely correct). The third question was specific to Mandarin as a tonal language, as it is possible to mispronounce a word in Mandarin by confusing one of the four lexical tones. In addition, we gave participants (4) a *Surprise Memory Test* at the end of the experiment, asking them to write down any of the learner’s utterances that they could recall from the video. This was included because past research has shown that iconic gestures help disambiguate audio-degraded speech (Obermeier et al., 2011; Drijvers and Özyürek, 2017), and it is possible that this disambiguation would manifest in recall for accented speech too.

##### Learner evaluation

To probe for different aspects of social impression about L2 learners, the following questions were presented: (5) *Confidence:* “How confident was this person?” (1 = not at all confident to 10 = extremely confident); (6) *Nervousness:* “How nervous was this person?” (1 = not at all nervous to 10 = extremely nervous); (7) *Communicative Effectiveness:* “How effective would this person be at communicating with native Mandarin Chinese speakers?” (1 = not at all effective to 10 = extremely effective); and (8) *Length of Study Time:* “How long do you think this person has been learning and practicing Mandarin Chinese?” [sliding scale labeled “amount of time (years)” from 0 to 20; it was converted to months later to be consistent with Experiment 2].

#### Procedure

The participants arrived at the Center for Language and Brain lab and were given a consent form. After they read the form, we clarified any questions before they signed it. The following script was read to participants of Experiment 1: “The purpose of this research is to evaluate the effectiveness of people speaking in Mandarin Chinese. You will view a series of brief videos of students practicing Mandarin Chinese, and after each one, take a survey to evaluate their learning efforts.” The intention of this introduction was to prime the participants to treat the L2 learners in the stimulus video as students, in addition to getting participants in the mindset of providing constructive feedback to L2 speakers.

After the basic introduction of the task, the researchers encouraged participants not to spend more than 1 min responding to all eight of the video’s questions. This time limit was introduced to emulate natural face-to-face communication in everyday life, during which listeners only have a very short time to process and integrate various sources of information about phonology, semantics, syntax, and pragmatics (Hanulíková et al., 2012). Participants were then brought into individual testing rooms, each containing of a computer, monitor and Pinyin keyboard on a desk.

The study was presented on Qualtrics. Participants were shown one video at a time, with each repeated twice. After that, the video would disappear from the screen, the set of seven survey questions appeared. The order of the questions, as described in the previous section, was set to a random order, and all participants answered them in this sequence: questions (5), (1), (2), (3), (8), (7), and (6). The experiment was self-paced, so the inter-stimulus interval length varied between participants. Each video and set of questions required about 45 s to 1 min. After participants finished responding to all the video stimuli, they were given the surprise memory test (question 4). The entire experiment lasted approximately 20–25 min.

After participants completed all of the tasks, the researcher debriefed them on the purpose of the study and compensated them with either course credit or $5 in cash.

#### Coding and Design

Aside from the rating scales, there were two measures that required coding: *Words Misheard*, with the question asking, “What did this person say,” and the *Surprise Memory Test* at the end.

The *Words Misheard* question was coded by comparing the participant’s typed answer to the actual speech in the video. A correct answer received a score of 0 (no errors), and an incorrect answer in any part of the utterance received a score of 1. The *Surprise Memory Test* involved free recall, and a score of “1” was given to phrases identical to the words presented in the study (complete memory) and a score of “0.5” was given to partially correct scores (partial memory), such as having the same root word but incorrect ending. A “0” was given for items that were entirely omitted or could not be traced back to any utterance (incomplete memory). In this way, low values for the “misheard” dependent variable (DV) mean better perception, whereas low values for the “memory” DV mean worse recall.

The experiment had a one-factor analysis of variance, with 3 conditions: culturally familiar gesture, culturally unfamiliar gesture, and speech alone.^3^ Because we make non-orthogonal comparisons among our three levels of condition, we used Dunn-Šidák multiple contrasts to correct for Type I errors.

The DVs were separated into two categories. First, the L2 “Speech” evaluation includes measurements concerning (1) Words Misheard, (2) Accent, (3) Tone Accuracy, and (4) Memory Test. Second, the L2 “Learner” evaluation includes measurements concerning (5) Confidence, (6) Nervousness, (7) Communicative Effectiveness, and (8) Judgments of Length of Time Studying Mandarin.

### Results

#### Speech Evaluation

Means and standard deviations of native Mandarin speaker responses are shown in Table 1. See the top half for the Mandarin data (see section “Experiment 1: Mandarin results).

**TABLE 1:** Means and standard deviations of native speaker responses in the L2 “Speech” evaluation.

	Words Misheard		Accent		Tone Accuracy		Memory Test	
	1 = misheard 0 = correct		10 = most native-like		10 = completely correct		100% = all words recalled	
	Mean	*SD*	Mean	*SD*	Mean	*SD*	Mean	*SD*
**Experiment 1: Mandarin**								
FAMILIAR gesture	0.04	0.09	5.62	1.19	6.60	1.12	42.4%	1.08
UNFAMILIAR gesture	0.09	0.10	5.21	1.24	6.25	1.31	44.9%	1.20
SPEECH alone	0.11	0.10	5.17	1.28	6.14	1.12	33.3%	1.10
**Experiment 2: Japanese**								
FAMILIAR gesture	0.01	0.04	5.03	1.19	N/A	N/A	17.9%	0.14
UNFAMILIAR gesture	0.02	0.05	4.83	1.31	N/A	N/A	17.8%	0.13
SPEECH alone	0.06	0.07	4.67	1.30	N/A	N/A	17.3%	0.12

**FIGURE 2:**
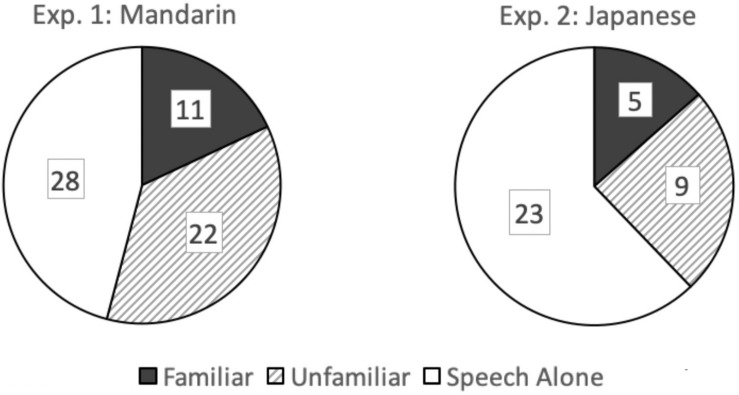
Number of words misheard in each of the familiar-gesture, unfamiliar-gesture, and speech-alone conditions.

For the proportion of misheard speech, there was a significant effect of gesture, *F*(2,70) = 5.065, *p* = 0.014, $\eta_{\text{p}}^{2}$ = 0.16. Familiar gestures produced lower error rates than both speech alone, tDS(3,35) = 2.757, *p* = 0.014, and culturally unfamiliar gestures tDS(3,35) = 2.743, *p* = 0.030. No significant difference was found between speech alone and unfamiliar gestures, tDS(3,35) = 1.03, n.s. The left panel of Figure 2 shows the number of Mandarin words misheard in each of the three conditions (out of a total number of 756 answers = 21 utterances × 36 native listeners). The figure clearly demonstrates that the familiar gesture condition yielded the smallest number of misheard words, contrasting with the unfamiliar gesture and speech alone conditions.

On the evaluation of accent, there was a significant effect of gesture, *F*(2,70) = 5.830, *p* = 0.005, $\eta_{\text{p}}^{2}$ = 0.143. Familiar gestures produced significantly more native-like ratings compared to speech alone, tDS(3,35) = 3.061, *p* = 0.006, and also compared to unfamiliar gestures, tDS(3,35) = 2.776, *p* = 0.014. However, there was no significant difference between unfamiliar gestures and speech alone, tDS(3,35) = 0.281, n.s. For tonal accuracy, there was a significant effect of gesture, *F*(2,70) = 4.206, *p* = 0.019, $\eta_{\text{p}}^{2}$ = 0.107. Familiar gestures influenced participants to attribute more correct tonal pronunciation than speech alone, tDS(3,35) = 2.791, *p* = 0.012. However, there were no significant differences between familiar gestures and unfamiliar gestures, tDS(3,35) = 2.085, n.s., or between unfamiliar and speech alone, tDS(3,35) = 0.670, n.s.

The surprise memory test also yielded a significant effect of gesture, *F*(2,70) = 5.045, *p* = 0.011, $\eta_{\text{p}}^{2}$ = 0.126, such that speech alone yielded worse recall than both culturally familiar, tDS(3,35) = 2.500, *p* = 0.026, and unfamiliar gestures, tDS(3,35) = 3.332, *p* = 0.006. However, there was no significant difference between familiar and unfamiliar gestures, tDS(3,35) = 0.552, n.s.

#### Learner Evaluation

Means and standard deviations of native speaker responses in the L2 “Learner” evaluations were given in the upper half of Table 2 (see section “Experiment 1: Mandarin results”).

**TABLE 2:** Means and standard deviations of native speaker responses in the L2 “Learner” evaluation.

	Confidence		Nervousness		Comm. Effectiveness		Months Studying*	
	10 = extremely confident		10 = extremely nervous		10 = extremely effective		0–50 months	
	Mean	*SD*	Mean	*SD*	Mean	*SD*	Mean	*SD*
**Experiment 1: Mandarin**								
FAMILIAR gesture	6.55	1.09	3.54	1.16	6.61	1.15	30.10	1.22
UNFAMILIAR gesture	6.65	1.19	3.55	1.09	6.42	1.15	29.67	1.23
SPEECH alone	6.17	1.29	3.91	1.16	6.13	1.17	27.67	1.38
**Experiment 2: Japanese**								
FAMILIAR gesture	7.15	1.05	2.96	1.42	6.84	1.03	15.72	6.66
UNFAMILIAR gesture	6.87	1.19	3.22	1.43	6.60	1.08	14.97	7.12
SPEECH alone	6.47	1.30	3.80	1.46	6.47	1.22	14.38	6.12

* The original data in ‘years’ for Mandarin experiment were converted to ‘months’ to match Japanese data.

For confidence, there was a significant effect of gesture, *F*(2,70) = 4.859, *p* = 0.011, $\eta_{\text{p}}^{2}$ = 0.122, with speech alone lowering confidence ratings compared to both familiar, tDS(3,35) = 2.214, *p* = 0.049, and unfamiliar gestures, tDS(3,35) = 3.049, *p* = 0.012. There was no significant difference between the familiar and unfamiliar gestures, tDS(3,35) = 0.646, n.s. For nervousness, there was a significant effect of gesture, *F*(2,70) = 3.311, *p* = 0.045, $\eta_{\text{p}}^{2}$ = 0.086. The mean rating appeared higher, i.e., more nervous, in speech alone than in the other conditions, as shown in Table 2. However, none of the individual comparisons yielded a significant difference [familiar gestures vs. speech alone: tDS(3,35) = 2.159, n.s.; familiar vs. unfamiliar gestures: tDS(3,35) = 0.028, n.s.; and unfamiliar vs. speech alone: tDS(3,35) = 2.059, n.s.]. (Note that finding null results with our planned contrasts, despite finding a significant omnibus effect in the ANOVA, is the result of using Dunn-Šidák multiple contrasts, which adjusted the criteria more strictly than without an adjustment).

For communicative effectiveness, there was a significant effect of gesture, *F*(2,70) = 6.644, *p* = 0.003, $\eta_{\text{p}}^{2}$ = 0.160. Both familiar and unfamiliar gestures were judged to be more effective than speech alone [tDS(3,35) = 3.240, *p* = 0.005; tDS(3,35) = 2.619, *p* = 0.039, respectively]. Between familiar and unfamiliar gesture, however, no significant difference was found, tDS(3,35) = 1.388, n.s.

For estimates of time studying the Mandarin language, there was no significant effect of gesture, *F*(2,70) = 1.457, n.s.

#### Experiment 1 Summary

##### Speech evaluation

The most consistent finding in the speech evaluation measures was that familiar gestures indicated an advantage over speech alone in all dimensions: with fewer words misheard, higher “native-like” accent ratings, higher tone accuracy, and more recalled utterances in the surprised memory test (see Table 3 for a summary of Experiment 1). However, effects of unfamiliar gestures were somewhere between the other two conditions—in two evaluations (tone accuracy and memory test), unfamiliar gestures did not differ from familiar gestures, but in the other two evaluations (words misheard and accent ratings) unfamiliar gestures showed significantly less advantage than familiar gestures. Compared with speech alone, unfamiliar gestures had only one advantage, producing more recalled items in the surprised memory test than speech alone, but they did not differ in the other evaluations. Our original prediction was that unfamiliar gestures would have a more negative effect than speech alone, but none of the cases showed this.

**TABLE 3:** Summary of significant differences between conditions: FAMILIAR Gesture, UNFAMILIAR Gesture, and SPEECH Alone.

		FAMILIAR vs. SPEECH	FAMILIAR vs. UNFAMILIAR	UNFAMILIAR vs. SPEECH
**(1) Speech evaluation**				
*Exp 1: Mandarin*	Words misheard	*	*	n.s.
	Accent	**	*	n.s.
	Tone	*	n.s.	n.s.
	Memory test	*	n.s.	**
*Exp 2: Japanese*	Words misheard	***	n.s.	*
	Accent	**	n.s.	n.s.
	Memory test	n.s.	n.s.	n.s.
**(2) Learner evaluation**				
*Exp 1: Mandarin*	Confidence	*	n.s.	*
	Nervousness	n.s.	n.s.	n.s.
	Comm. Effectiveness	**	n.s.	*
	Months studying	n.s.	n.s.	n.s.
*Exp 2: Japanese*	Confidence	***	n.s.	***
	Nervousness	***	*	***
	Comm. Effectiveness	**	*	n.s.
	Months studying	*	n.s.	n.s.

* p < 0.05, ** p < 0.01, *** p < 0.001. In all cases, the direction of differences was more positive evaluation (e.g., less words misheard, more words memorized, more confidence, and less nervousness) for FAMILIAR than SPEECH, FAMILIAR than UNFAMILIAR, and UNFAMILIAR than SPEECH.

##### Learner evaluation

Two major patterns were found for evaluation of L2 learners. First, we found that familiar and unfamiliar gestures both led to higher ratings of confidence and communicative effectiveness, compared to speech alone. In contrast, for the evaluation of nervousness and the estimate of time studying Mandarin, there were no differences among the three conditions.

## Experiment 2: Japanese

Experiment 2 attempted to build on Experiment 1 by generalizing to a different language and culture: Japanese. Given that the vast majority of research in psychology has focused on Western societies and English speakers, it is important to increase diversity in the field by expanding to different cultures and languages (Henrich et al., 2010). It goes without saying that there are vast differences among Asian languages and cultures as well. This diversity is especially relevant for the topic of emblematic gestures, which by definition depend on the specific conventions of a particular culture.

Combined with the authors’ impressions and discussions with native Chinese and Japanese speakers, we reasoned that these two cultures might vary to different degrees in the use of gesture, making Japanese emblems a good candidate for the present study.

More importantly, we considered another point: The Mandarin speakers in the first experiment were enrolled in a university in the U.S. for 0.5–3.5 years at the time of testing, and they were proficient in English for undergraduate studies. This factor might have exposed them to a greater variety of linguistic and cultural elements outside their native language and culture, and it might have made them more open to difference than people who have never lived abroad. With this in mind, we sought to find college students in Japan who did not have as extensive experience abroad. This may make the interpretation of emblems relatively more uniform across these participants in Japan, which would be a nice contrast with the Chinese participants in Experiment 1.

Using the same basic paradigm as Experiment 1, we investigated the extent to which native Japanese speakers are sensitive to the cultural meaning of emblem gestures when: (1) perceiving non-native speech and (2) forming social impressions of non-native speakers.

### Method

The method for Experiment 2 was largely borrowed from Experiment 1 with a few notable differences that will be addressed in this section.

#### Participants

Forty-eight native Japanese undergraduate college students (all females) from a small all-women’s college in Tokyo participated in Experiment 2. All participants had limited exposure to the English language, mostly having learned it formally in school. None of them had experience of studying abroad for more than a year. Participants received 1,000 yen, the rough equivalent of ten U.S. dollars, for their participation.

#### Materials

##### L2 learner stimuli

There were thirty “learners of Japanese” who were students of a small liberal arts university on the East Coast in the U.S. Similar to Experiment 1, learners represented varying levels of accented Japanese speech. Learners ranged from no exposure to students who had been learning the Japanese language for 3 or 4 years (400-level). Learners on the video represented a wide spectrum of racial, ethnic and gender diversity.

##### Video clips

**FIGURE 3:**
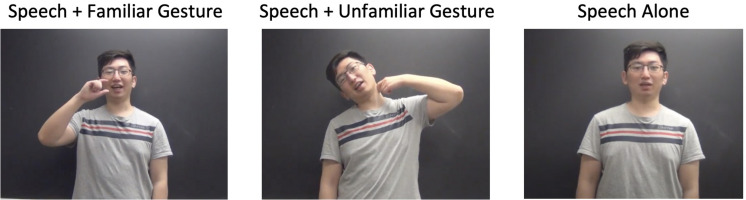
Stimuli example from Experiment 2: Go for a drink? (“Nomi ni ikanai”?).

The process of creating the 30 video clips was the same as described in Experiment 1. Each stimulus was assigned a short Japanese phrase. For example, consider the Japanese phrase for saying “Go for a drink?,” which was “Nomi ni ikanai?” In Japanese, a culturally familiar emblem for “Go for a drink?” is a two-finger gesture with the index finger and thumb positioned horizontally, tilting toward the mouth. To create the culturally unfamiliar condition, the same speech would be paired with a Russian gesture for “Go for a drink?”: a tilt of the head with a light flick on the side of one’s neck. As a baseline, the third condition was the learner speaking in the video without any gesture. Each learner was first instructed to perform in these three conditions and then videotaped. See Figure 3.

After all the videos were created, there were some concerns that a few of our gesture-speech pairs were not as good as others. To explore this possibility, we ran a “norming” test asking four native Japanese speakers to evaluate the cultural familiarity of our emblems in each of our familiar-unfamiliar pairs across all 30 items. Specifically, the four native Japanese speakers first read the Japanese phrase, and then viewed each of the gestures, familiar and unfamiliar, paired with that phrase. For each phrase, they were asked to keep it in mind and judge how naturally the gesture captured that meaning in the scale of 1 (not at all natural) to 10 (completely natural). Based on this norming study, it was discovered that two items had a pattern of rating in which the familiar and unfamiliar emblems were rated as very close to one another and two items in which the familiar gestures were rated as *less natural* than the unfamiliar gestures. Consequently, these items were eliminated from all analyses presented below. This removal did not change the significance of the results, except for the question about how long learners had been learning Japanese. The total of 26 stimuli used in the final analysis were shown in Appendix 3 Supplementary Materials.

#### Evaluation of Learners’ Videos

The questionnaire was very similar to Experiment 1, with two sets of questions focusing on (1) speech and (2) social impressions of learners, except that it was given in Japanese, the participants’ L1 in Experiment 2. There were four minor changes in the wording of a few of the questions. First, in Experiment 1, the scale representing accentedness read: 1 (completely foreign) to 10 (completely native). In Experiment 2, this was changed to: 1 (not at all native) to 10 (completely native) to maintain consistency within the vocabulary used. Second, another question in Experiment 1 asked the participants to estimate how long the learner “has been learning and practicing Mandarin Chinese” on a scale ranging from 1 to 20 years. This scale did not seem to be very effective, as the mean score for each condition displayed around 2 years of perceived learning. For Experiment 2, we altered the scale to 1 – 50+ months which was labeled on a sliding scale. Third, while in Experiment 1 the scale measuring nervousness read 1 (not at all nervous) to 10 (extremely nervous), Experiment 2’s nervousness scale was inverted: 1 (extremely nervous) to 10 (not at all nervous). To be consistent with Experiment 1, we converted the scores from Experiment 2 to the scale in Experiment 1, in which higher numbers mean *more* nervous. And fourth, the question relating to tone in Experiment 1 was removed for Experiment 2, given the difference in the use of fundamental frequency in Japanese and Chinese phonology (Howie and Howie, 1976; Vance, 2008).

#### Procedure

The basic procedure was the same as Experiment 1, but the instructions were given in Japanese by one of the two experimenters who spoke advanced Japanese. The testing site was also different from Experiment 1 because Experiment 2 took place at a small all women’s college in Japan. Time slots for the study were set up so that 2 participants would come for the study at the same time. The testing room was set up so that the tables lined the perimeter of the room. Participants sat in the two corners, each setup with a laptop and headphones, facing the same wall so that the researchers could see when they finished. The study took about 45 min to complete. The experimenters waited until both participants were done, and they were debriefed together in Japanese at the end.

#### Coding, Design, and Analyses

We used the same basic design as Experiment 1, which was a one-factor analysis of variance, with condition (3 levels) as a within-subjects factor. The open-ended questions (*words misheard* and *memory test*) were coded in the same way as Experiment 1.

### Results

Means and standard deviations of native Japanese speaker responses are shown in Tables 1, 2. See the bottom half of each table for the Japanese data.

#### Speech Evaluation

For the proportion of misheard speech, there was a significant effect of gesture, *F*(2,94) = 8.076, *p* = 0.001, $\eta_{\text{p}}^{2}$ = 0.147, with speech alone producing higher proportions of errors than both familiar gestures, tDS(3,47) = 3.766, *p* < 0.001, and unfamiliar gestures tDS(3,47) = 2.615, *p* = 0.036. There was no difference between unfamiliar and familiar gestures, t(3,47) = 1.077, n.s. The right panel of Figure 2 shows the number of Japanese words misheard in each of the three conditions (out of a total number of 1,248 answers = 26 utterances × 48 native listener participants). Although the total number of misheard words was quite small, it is notable that roughly 60% of the errors occurred in the speech alone condition.

There was a significant effect of gesture on accent perception, *F*(2,94) = 4.980, *p* = 0.010, $\eta_{\text{p}}^{2}$ = 0.096, with familiar gestures producing higher native-like ratings than speech alone, tDS(3,47) = 3.087, *p* = 0.005. However, unfamiliar gestures did not significantly differ from familiar gestures, tDS(3,47) = 1.978, n.s., and from speech alone, tDS(3,47) = 1.293, n.s.

For the surprise memory test, there was no significant effect of gesture, *F*(2,94) = 0.033, n.s.

#### Learner Evaluation

For confidence, there was a significant effect of gesture, *F*(2,94) = 13.645, *p* < 0.001, $\eta_{\text{p}}^{2}$ = 0.225. Familiar gestures produced significantly higher confidence ratings than speech alone, tDS(3,47) = 4.690, *p* < 0.001. In addition, unfamiliar gestures produced higher scores than speech alone, tDS(3,47) = 3.672, *p* < 0.001. However, scores did not differ between familiar and unfamiliar gestures, tDS(3,47) = 2.045, n.s.

For nervousness, a significant effect of gesture was also found, *F*(2,94) = 20.310, *p* < 0.001, $\eta_{\text{p}}^{2}$ = 0.302. Familiar gestures produced lower nervousness ratings than both speech alone, tDS(3,47) = 5.825, *p* < 0.001, and unfamiliar gestures, tDS(3,47) = 2.328, *p* = 0.036. In addition, unfamiliar gestures produced lower nervousness ratings than speech alone, *t*(3,47) = 3.971, *p* < 0.001.

For communicative effectiveness, there was also a significant effect of gesture, *F*(2,94) = 5.725, *p* = 0.006, $\eta_{\text{p}}^{2}$ = 0.109, with familiar gestures judged as more effective than both speech alone, tDS(3,47) = 2.888, *p* = 0.009, and unfamiliar gestures, tDS(3,47) = 2.354, *p* = 0.035. However, there was no difference between unfamiliar gestures and speech alone, tDS(3,47) = 1.254, n.s. For estimates of time studying the Japanese language, a significant effect of gesture was also found, *F*(2,94) = 3.146, *p* = 0.048, $\eta_{\text{p}}^{2}$ = 0.063. Learners with familiar gestures were judged as studying the language longer than those with speech alone, *t*(3,47) = 2.456, *p* = 0.027. However, no other comparisons yielded significant differences.

#### Experiment 2 Summary

##### Speech evaluation

Table 3 presents a summary of Experiment 2. Familiar gestures were associated with less mishearing and higher ‘native-like’ accent ratings than speech alone, showing their advantage. Interestingly, familiar gestures did not differ from unfamiliar gestures in both of these two evaluations. Unfamiliar gestures showed one advantage over speech alone, having less misheard words. Unlike Experiment 1, there were no differences across conditions in recall accuracy for the memory test.

##### Learner evaluation

Positive effects of familiar gestures were robust in the learner evaluation: Familiar gestures were associated with more confidence, less nervousness, more effectiveness in communication, and judgments of longer months of study than speech alone. In addition, familiar gestures were more advantageous than unfamiliar gestures in two of the four evaluations as well (less nervous and more effective in communication), but not in the other evaluations. Just as in Experiment 1, effects of unfamiliar gestures were somewhere between the other two conditions: Unfamiliar gestures produced higher confidence ratings and lower nervousness ratings than speech alone, but the two conditions did not differ in the other two evaluations.

## General Discussion

### Culturally Familiar Gestures Help, Uniformly

The results from the two experiments, as summarized in Table 3, provide strong support for our first prediction. We predicted that, relative to speech alone, culturally familiar gestures would improve speech perception and memory, as well as social impressions of the L2 learner (Popelka and Berger, 1971; Gullberg, 1998; Gregersen, 2005; Kelly et al., 2009; Maricchiolo et al., 2009).

In Experiment 1, we found that familiar gestures produced more positive responses than speech alone in all of the *speech* evaluation dimensions: fewer perception errors, higher “native-like” accent ratings, higher tone accuracy, and greater words recalled in the surprise memory test. Similarly, Experiment 2 revealed that familiar gestures produced fewer perception errors and higher accent ratings compared to speech alone (but, unlike Experiment 1, such benefit was not observed in the memory test).

These advantages of familiar gestures over speech alone extend to include the social impression of L2 *learners*. Culturally familiar gestures raised ratings in two of the four evaluations—confidence and communicative effectiveness—in Experiment 1, and in addition, they positively affected all of the evaluations in Experiment 2, including the lower judgments of nervousness and higher estimates of how long learners had been studying Japanese. The findings that familiar gestures positively influenced speech perception is consistent with literature showing that semantically related speech and gesture improve accuracy of L1 comprehension (Popelka and Berger, 1971; Graham and Argyle, 1975; Kelly et al., 2010; Dahl and Ludvigsen, 2014) and vocabulary retention in L2 learning (Allen, 1995; Sueyoshi and Hardison, 2005; Sime, 2006; Kelly et al., 2009; Morett, 2014), in addition to boosting speech perception when auditory information is moderately degraded (Obermeier et al., 2011; Drijvers and Özyürek, 2017). Adding to this work, the present study demonstrates that the *cultural* relationship between L2 speech and gesture matters, too. When gestures culturally match the L2—what we call, culturally familiar emblems—they play a positive role in shaping how L2 speech is perceived. Moreover, going beyond previous work by Allen (1995), our results show that not only is the mere presence of emblematic gestures useful, but their specific cultural content matters, too.

Focusing first on perception errors, what mechanism might explain why culturally familiar gestures best help native speakers to hear speech correctly? Considering Experiment 1, there were not many instances of misheard speech across the board (about 8%), but familiar gestures were particularly low with only a ∼4% error rate. In contrast, unfamiliar gestures more than doubled that rate (∼9%) and having no gestures produced even more errors (∼11%). In Experiment 1, familiar gestures also boosted judgments of Mandarin tonal pronunciation accuracy. One possibility is that because culturally familiar gestures are so easily recognizable for native speakers, it may have required minimal cognitive effort to process their meaning, leaving adequate perceptual resources to focus on the L2 speech (Adank et al., 2009).

With regard to accent ratings, the results from both experiments add to the literature on the phonological functions of co-speech gesture. While previous research has shown that the hand movements of speakers—in an L1 (Krahmer and Swerts, 2007; Pouw et al., 2020) and L2 (Gluhareva and Prieto, 2017; Zheng et al., 2018; Hoetjes et al., 2019)—affect perceptions of speech by L1 users, no study to our knowledge has shown that *viewing* culturally familiar gestures can modulate how non-native accents are perceived by native speakers. In both experiments, we show that the presence of culturally familiar gestures improves ratings of accentedness compared to no gestures. Previous research has shown that contextual factors, such as race of speaker (Jussim et al., 1987; Rubin, 1992; Hansen et al., 2017), can modulate perception of accent; here, we extend this phenomenon to include not just these fixed features of the context, as in the case of speaker identity, but more fluid factors, such as what people do with their hands. This fits well with research on the processing of speech in the context of other dynamic multimodal signals, such as the integration of facial expressions, body posture and emotional tone of voice (Pourtois et al., 2005; Van den Stock et al., 2007).

The benefits of producing culturally familiar gestures also extend to managing social impressions of others. Consistent with our first prediction, we found that, for both experiments, the presence of familiar gestures led to more positive impressions than speech alone. This work fits nicely with previous studies on the social benefits of co-speech gesture for L1 (Maricchiolo et al., 2009) and L2 speakers (Gullberg, 1998; Gregersen, 2005). For example, Gullberg (1998) found that native speakers made more positive evaluations of L2 learners who produced many iconic gestures. And with regard to nervousness, Gregersen (2005) showed that native speakers judged L2 learners to be more at ease when they used many emblematic and iconic gestures, and in contrast, more anxious when they produced mostly non-communicative self-adaptors (e.g., fidget with objects, touching face and hair, adjusting clothing) or no gestures at all (e.g., hands in lap or arms crossed). Moreover, all of these studies focused on Indo-European languages and learners from the US and Europe, and we extend beyond that by adding data from non-Indo-European languages, Mandarin and Japanese, and a different part of the world, Asia.

It is worth adding that in Experiment 2, culturally familiar gestures helped social impressions more than unfamiliar ones (Table 3). Specifically, familiar gestures lowered assessments of nervousness and improved judgments of communicative effectiveness for the Japanese viewers. This suggests that at least some of the time, simply waving the hands is not enough to make a good impression—the cultural meaning of gesture matters. It is interesting that this pattern held only for the native speakers of Japanese, but not Mandarin. One possible reason for this inconsistency is that the culturally familiar and unfamiliar gestures were better differentiated for Japanese native speakers in Experiment 2. This could be due to there being more consistency and uniformity of emblems in Japan and more variability of emblems in China. Another possibility is that because we tested Chinese international students—who were attending college abroad in the United States with other international students—it could be that they simply had been exposed to a wider a diversity of emblems. This cultural exposure may have made them more open-minded to “unfamiliar” gestures and ultimately diluted the difference between the two conditions. We will return to the differences between our two samples in a later section. It is also possible that norming familiar and unfamiliar emblems in Experiment 2, but not in Experiment 1, contributed to the different findings for social impressions between the two experiments.

### Culturally Unfamiliar Gestures Help, Variably

Our second prediction was that, relative to speech alone, culturally *unfamiliar* gestures would decrease foreign accent ratings and encoding/memory accuracy of L2 speech (Popelka and Berger, 1971; McNeill et al., 1994; Kelly et al., 2009), in addition to lowering social impressions of the accented speaker. Our results in the two experiments indicated that this prediction was not supported at all. In no cases did unfamiliar gestures produce significantly more negative responses than speech alone in the evaluations of L2 speech and L2 learners. Instead, unfamiliar gestures produced more advantageous ratings than speech alone in some evaluation questions (the right-most column in Table 3). For example, compared to speech alone, unfamiliar gestures were associated with greater number of recalled words in the Mandarin experiment, and with fewer misheard words in the Japanese experiment, while other evaluations yielded no difference between the two conditions. For the social evaluation of L2 learners, unfamiliar gestures, compared to speech alone, were associated with more confidence and higher communicative effectiveness in the Mandarin study, and with more confidence and less nervousness in the Japanese study.

We also found a surprising result that, in the majority of evaluation questions, unfamiliar gestures did not differ from familiar gesture (see the middle column in Table 3). For example, none of the evaluation questions showed a difference between familiar and unfamiliar gestures in the social impression of the Mandarin learners, and also there were no differences in speech evaluations of the Japanese learners. This pattern was unexpected given that native speakers have difficulty processing non-native speech under adverse listening conditions (Adank et al., 2009; Bent and Atagi, 2017). From that perspective, we expected unfamiliar emblems to distract native speakers, depleting their perceptual resources, which would cause them to make more encoding errors than the optimal familiar gesture condition—but that was not the case. What might be going on?

A prominent framework for research on multimodal communication is Clark and Paivio’s dual coding theory of information processing (Paivio, 1990; Clark and Paivio, 1991). By this traditional account, communication is enhanced when there is both a verbal and imagistic channel, and this is theorized to be the case even when the two channels do not convey the same semantic content. Although most gesture researchers treat the semantic relationship between speech and gesture as critical, there is some evidence that semantic congruence is not always essential. For example, even beat gestures, which often have little inherent semantic connection to speech, affect L1 speech processing (Krahmer and Swerts, 2007; Biau and Soto-Faraco, 2013; Wang and Chu, 2013) and memory (So et al., 2012). And in an L2 context, there is evidence that viewing and producing a range of hand movements—beat gestures (Kushch et al., 2018), metaphoric pitch gestures representing lexical tone (Morett and Chang, 2015; Baills et al., 2019) and even iconic gestures with idiosyncratic meanings (Macedonia and Klimesch, 2014; Huang et al., 2019)—can help with L2 vocabulary learning and retention. Connecting these findings to the present study, it is interesting that the presence of any gesture increased memory for speech in Experiment 1 (both gesture conditions produced a ∼30% improvement in recall over speech alone) and decreased the number of “misheard” utterances in Experiment 2 (both gesture conditions reduced errors by over 60% compared to speech alone). This suggest that at least on occasion, the mere act of moving the hands as a non-native speaker may help draw attention to the accompanying speech, much like a beat gesture functions, while also providing a visual anchor to help listeners remember what was said—no matter the meaning of the gesture.

For social impressions of the learners, the presence of any type of emblem also seemed to have some benefits. It is possible that our gestures, even when they culturally missed the mark, functioned to signal social effort, which may have led participants to evaluate learners who gestured in a more positive light (Gullberg, 1998; Maricchiolo et al., 2009). In the case of the Japanese experiment, perhaps not gesturing at all was a sign of anxiety when speaking the L2, whereas simply moving the hands to *intentionally* communicate anything—no matter whether it was culturally appropriate—signaled that L2 learners were more at ease (Gregersen, 2005).

Finally, it is worth considering the possibility that our participants did not always view our “culturally unfamiliar emblems” as emblems *per se*. Perhaps they occasionally viewed them as regular co-speech iconic gestures, albeit unusual and obscure ones. Because co-speech iconic gestures are not bound by conventional standards as much as emblems, and because many of the unfamiliar emblems in the two experiments had distinct iconic properties, native speakers may have given some of the unfamiliar gestures much more leeway when produced by L2 speakers. This highlights the important issue of variability, which we discuss next.

### Variability

Traditionally, finding variability in results across samples and within conditions is seen as a red flag, an indication of weak external and internal validity. However, we see it differently in the present study. For one, collecting diverse samples of participants intentionally opens the door to more variability (Henrich et al., 2010). Beyond the diversity of studying non-native English speakers from Asia, we also had important differences between our two samples: In Experiment 1, we studied native Mandarin speakers who were fluent in English and attended college in the United States, whereas in Experiment 2, we studied native Japanese speakers who were mostly monolingual and had not spent extended periods outside of Japan. These differences are sure to cause some variation in the results.

Looking at the comparison between familiar and unfamiliar gestures in the learner evaluation (the middle column of Table 3), unfamiliar gestures were associated with disadvantage in none of the social impression questions for the Mandarin Chinese participants. This contrasts with the Japanese participants showing a disadvantage of unfamiliar gestures in two of the four social impression questions. One possibility is that participants in our Mandarin sample may have been exposed to a wider diversity of emblems, both in China with its diversity of cultural gestures (based on its higher linguistic diversity) and in America on a college campus with students and faculty from dozens of countries from around the world. This exposure might have contributed to Chinese participants more generously appreciating the speakers’ effort than the Japanese participants’ exposure mostly to their domestic gestures.

Another source of variability comes from the diverse functions of gestures themselves (Church et al., 2017; Novack and Goldin-Meadow, 2017). For example, Novack and Goldin-Meadow (2017) point out that gestures play multiple roles across contexts—communicating, problem solving, learning and remembering—and across social roles—for those who *view* gesture and those who *do* gesture. The present study taps into a wide range of these multiple functions: L2 learners produced emblems while communicating foreign utterances, and then native speakers viewed those gestures to perceive and recall speech, form judgments about non-native accents, and make social assessments about communicative effectiveness, confidence and nervousness. Given these varied functions, it makes sense that the cultural familiarity of gesture may at times be important in social categorization, but not in perceptual processing. And at other times, it is not surprising that it is the other way around.

Finally, one limitation of our study is that although it was well powered to run subject analyses, it was not adequately powered to run item analyses. Still, we did run unofficial item analyses on eleven of our fifteen dependent measures across both experiments. In our lower powered experiment (Experiment 1, which had 21 items), five of our significant effects were lost, but in our higher powered experiment (Experiment 2, which had 26 items), only one effect was lost. Interestingly, in each experiment, there was one new significant effect. Because these were underpowered analyses, it is hard to interpret them: On one hand, it could be that there indeed was more variability across items than subjects; but on the other hand, the variability could actually be comparable, but because there were far fewer items than subjects, the statistical differences among conditions was diluted in the item analyses. Following up on this, if increasing the number of items produces similarly robust effect sizes as the present study, it would strengthen our conclusion that co-speech emblems plays a beneficial role in cross-cultural contexts.

### Future Studies

It is worth noting that there is another function of gesture that was intentionally missing from the present study, but likely would have also played a major role. Recall that producing gestures affects vocal production in an L1 (Krahmer and Swerts, 2007; Pouw et al., 2020) and L2 (Gluhareva and Prieto, 2017; Zheng et al., 2018; Hoetjes et al., 2019). In the present study, we dubbed identical speech onto each of our three video conditions in order to control for this vocal function of gesture. However, in the wild, this vocal effect of gesture runs free. This means that there may be layered roles of cultural emblems: not only would they function to visually influence the way spoken information is processed and evaluated by others (as we have shown), but they may also directly affect the quality of the actual speech signal itself. Going forward, it would be interesting to move beyond showing that culturally appropriate gestures positively influence how non-native speech is *received* and also explore whether a gesture’s cultural appropriateness affects how non-native speech is actually *produced*. Does asking, “Nomi ni ikanai?,” with the *right* drinking emblem help a learner vocally articulate that Japanese utterance any better? This is an interesting question to pursue in the future.

Even if producing appropriate emblems does not actually help learners pronounce L2 speech, it could make them *believe* it does. Consider that in a recent study by Zheng et al. (2018), novice L2 speakers of Mandarin self-reported that making the gestures corresponding to lexical tones was vastly more helpful in pronouncing the tones than not gesturing at all. And anecdotally, during the filming session of our study, many of the L2 learners informally commented that their pronunciation felt the best when they produced gestures. In this way, producing emblems—culturally right or wrong—may serve multiple and varied purposes in cross-cultural communication, and future work should attempt to disentangle these diverse functions.

### Theoretical and Practical Implications

Starting with David McNeill’s seminal 1985 paper, *So you think gestures are nonverbal*, there has been growing interest in understanding gesture and speech as an integrated semiotic system, as a window into the mind of a speaker. Indeed, many of the papers in this *Frontiers Research Topic* are focused on mental aspects of this integrated system of meaning. However, as Kendon (2017) recently pointed out, this focus on the cognitive components of gesture—while extremely valuable—has often eclipsed the many potent social function of the hands (see also Church et al., 2017). Kendon reminds us that gestures also have a distinct cultural component (Kendon, 1997), and together with speech, the two modalities combine to create a powerful pragmatic tool (Kendon, 2017). And if this is the case for one’s L1, it may apply doubly for wielding a second language. Recall that Gullberg (2006) makes a strong case that mastering an L2 gesture repertoire is key to a learner’s “cultural fluency.” Indeed, as far back as Efron (1941), we have known that gestures signal social identity and that learning to adapt them to new contexts and environments is a sign of successful cultural assimilation.

This cultural component of hand gesture has been absent in research on the social stigma of non-native accents (Giles, 1977; Gluszek and Dovidio, 2010; Lev-Ari and Keysar, 2010; Kinzler et al., 2011; Lippi-Green, 2012; DeJesus et al., 2017). This is noteworthy because although relatively fixed aspects of one’s identity (e.g., gender, race and class) are well known to affect how accents are received (Jussim et al., 1987; Rubin, 1992; Van Berkum et al., 2008; Hansen et al., 2017), there has been much less attention to how more fleeting aspects of context influence accent perception and evaluation. What speakers do with their bodies is a ubiquitous, but fluid and ever-changing, part of the way one speaks a native or non-native language. Focusing specifically on non-native accents, we have shown that this dynamic gestural context can affect many different aspects of how native speakers receive accented speech: correctly or incorrectly hearing and remembering what was said; positively or negatively shifting evaluations of pronunciation; increasing or decreasing impressions of confidence and nervousness; and raising or lowering judgments of communicative and cultural competence.

Bridging these two lines of work—research on accent and research on gesture—opens up new and important practical and theoretical questions. For example, how does what you do with your hands interact with more stable features of one’s identity, like race, gender or class? Because non-native accents are so hard to change beyond the sensitive period (Johnson and Newport, 1989), might gesture be used as a compensatory tool to give speech a hand? Given that traditional L2 instruction typically focuses on teaching correct *spoken* language (Jungheim, 2001), could this instruction be improved by also teaching students how to correctly gesture more systematically and comprehensively? This is an exciting question since it may bear on a learnable element that gives everyone a chance to improve, contrasting with one’s fixed social identity or hard to change non-native accent.

## Conclusion

To our knowledge, no previous study has explored the combined perceptual and social benefits of co-speech emblems in L2 communication. The results from our two experiments suggest that, during cross-cultural communication, visual information conveyed through hand gesture influences low level phonetic perception, in addition to higher level social evaluation. We have shown that perception and evaluation improve when L2 speakers use emblems—both culturally familiar and unfamiliar—even if non-native accents themselves stay the same and even when it spans very short utterances of a few seconds. This suggests that in cross-cultural communication, more attention should be paid to what L2 learners do with their hands.

## Data Availability Statement

The raw data supporting the conclusions of this article will be made available by the authors, without undue reservation.

## Ethics Statement

The studies involving human participants were reviewed and approved by the Colgate University, Internal Review Board. The participants provided their written informed consent to participate in this study. Written informed consent was obtained from the individual(s) for the publication of any potentially identifiable images or data included in this article.

## Author Contributions

SK, YH, and KB-V: question formation. KB-V and ZL: stimulus creation and data collection. SK, YH, KB-V, and ZL: coding, transcription, and analysis. KB-V: writing first draft. SK and YH: writing later drafts. YH: figures and tables. All authors contributed to the article and approved the submitted version.

## Conflict of Interest

The authors declare that the research was conducted in the absence of any commercial or financial relationships that could be construed as a potential conflict of interest. The handling editor declared a past collaboration with one of the authors SK.

## Footnotes

1 Of course, not all emblems have clear iconic meanings (e.g., the thumbs up gesture and OK sign mean different things across different cultures). Moreover, in other cases, the original iconic meanings become more obscure over time (e.g., it is believed that crossing the fingers for good luck is a vestigial iconic reference to the Christian cross) (Matsumoto and Hwang, 2013).

2 https://www.gestpedia.com/

3 All of the analyses presented in both experiments used subjects as the error term. Ideally, we would have liked to also run parallel ANOVAs with item as the error term, but because we had far fewer items than subjects, our design was too underpowered to draw valid conclusions for item analyses. We address this limitation in the section “General Discussion.”
