# Supplementary material for: Emblem Gestures Improve Perception and Evaluation of Non-native Speech
Source: Front Psychol. 2020 Sep 22;11:574418. doi: 10.3389/fpsyg.2020.574418 (PMC7536367; doi:10.3389/fpsyg.2020.574418)
Supplement: Supplementary file 1 [file Table_1.docx]

**Appendices**

Appendix 1. Experiment 1 Mandarin Study: Video Content

| **Phrase** | | | **Gestures** | |
| --- | --- | --- | --- | --- |
| **Meaning** | **汉字** | **Pinyin** | **Culturally Familiar** | **Culturally Unfamiliar** |
| I'm full | 吃得好饱 | chī dé hǎo bǎo | [Tap stomach with palms](https://www.gestpedia.com/videos/241/wo-bao-le/) | [*Stroke thumb across throat*](https://www.gestpedia.com/videos/348/syt-pa-gorlo/) *(Russia)** |
| No (I don't want) | 不用了 | bùyòngle | [Wave hands (palm open, facing front) in front of body](https://www.gestpedia.com/videos/239/bui-yao/) | *Wave hands (palm facing side) back and forth in front of body (Japan)* |
| It's 6 dollars | 六块钱 | liù kuài qián | [Thumb and pinky finger straightened while other fingers are folded (palm inwards)](https://pbs.twimg.com/media/BQwZCtGCcAATPUB.jpg) | [*5 fingers on one hand (palm open), 1 finger from other hand laying on top*](https://d20aeo683mqd6t.cloudfront.net/images/imgs/000/004/176/medium/2326.jpg?1528708884) *(Japan)* |
| It's 7 dollars | 七块钱 | qī kuài qián | Thumb, index, and middle fingers straightened, touching, and pointed forwards with other two fingers folded | [*5 fingers on one hand (palm open), 2 fingers from other hand laying on top*](https://d20aeo683mqd6t.cloudfront.net/images/imgs/000/004/177/medium/2327.jpg?1528708892) *(Japan)* |
| It's 8 dollars | 八块钱 | bā kuài qián | Thumb and index fingers straightened with the other three fingers folded, wrist at a right angle, palm facing inwards | [*5 fingers on one hand (palm open), 3 fingers from other hand laying on top*](https://d20aeo683mqd6t.cloudfront.net/images/imgs/000/004/178/medium/2328.jpg?1528708899) *(Japan)* |
| It's 9 dollars | 九块钱 | jiǔ kuài qián | Fist with index finger making a hook | [*5 fingers on one hand (palm open), 4 fingers from other hand laying on top*](https://d20aeo683mqd6t.cloudfront.net/images/imgs/000/004/179/medium/2329.jpg?1528708908) *(Japan)* |
| It's 10 dollars | 十块钱 | shí kuài qián | One index finger crossing with the other at a right angle, resembling a cross | *Both hands with all fingers open, palm facing front (Japan)* |
| Please be quiet | 请安静一下 | qǐng ān jìng yī xià | [One hand flat, palm down resting on an index finger point](https://youtu.be/30xHcRKFgv0?t=38s) | *Hold hand in front of body (USA)* |
| Sorry | 对不起 | duì buqĭ | [Both hands slightly folded over and framing eyebrows](https://youtu.be/Vn48HwWV2Fg?t=1m15s) | *Full hand, palm inwards, placed on chest (near heart) (USA)* |
| Thank you | 谢谢 | xièxie | Hand put together like praying | [*Tap right palm on chest*](https://www.gestpedia.com/videos/424/shukuran/) *(Egypt)* |
| Show respect for someone | 久闻大名 | jiǔ wén dà míng | [Right fist clasps into the palm of left hand](https://youtu.be/0SpW7CNJ4Do?t=1m30s) | *Salute (USA)* |
| I'm in trouble | 好烦啊 | hǎo fán a | Hands laced behind head | *Scratching back of head (Japan)* |
| Okay | 好啊 | hǎo a | Thumb & index fingers making a circle while other three fingers are straightened | [*Make a circle above head with both hands*](https://www.gestpedia.com/videos/467/ok/) *(Japan)* |
| It's spicy (food) | 好辣 | hǎo là | Wave hand in front of mouth (it can be either tongue out or open the mouth a little bit) | [*Hold top of nose bridge*](https://www.gestpedia.com/videos/451/shimiru/) *(Japan)* |
| I'm angry | 我很生气 | wǒ hěn shēngqì | Hand in a fist, raised arm to form roughly 90^o^ angle | [*Two index fingers behind head*](https://files.tofugu.com/articles/japan/2014-01-30-japanese-body-language/japanese-body-language-angry-finger-horns.jpg) *(Japan)* |
| Hmm, I don't think so | 我不这么认为 | wǒ bù zhè me rèn wéi | Shake head side to side | *Tilt head sideway (Japan)* |
| Are you talking to me? | 你在跟我说话吗？ | nǐ zài gēn wǒ shuō huà ma | Point to face | *Point towards + touch nose (Japan)* |
| I don't know | 我不知道 | wǒ bù zhīdào | Shrug shoulders | [*Shake head, point to self, wave as you cross pointed fingers*](https://www.gestpedia.com/videos/48/i-don-t-know/) *(Nigeria)* |
| Good luck | 祝你好运 | zhù nǐ hǎo yùn | Thumbs up | *Crossing index & middle finger (USA)* |
| Hurry up | 快一点 | kuài yīdiǎn | One hand making a fist with palm down, other hand pointing towards wrist | *Rub thumb and index (Mexico)* |
| I'll keep this a secret | 我不会跟人说的 | wǒ bù huì gēn rén shuō de | Drag hand in front of mouth while bringing thumb and index together like a zipper | [*Place index finger on lips*](https://www.gestpedia.com/videos/470/himitsu/) *(Japan)* |

* The country in the parenthesis shows where the gesture is often, but not exclusively, used.

Appendix 2. Experiment 1 Mandarin Study: Stimuli Distribution by Test Version

|  | **Version A** | **Version B** | **Version C** |
| --- | --- | --- | --- |
| Culturally Familiar Gesture | Hurry up | It costs 8 dollars | I’ll keep this a secret |
|  | Thank you | Are you talking to me? | It’s spicy |
|  | Good luck | Okay | I don’t know |
|  | I’m in trouble | It’s 10 dollars | I’m angry |
|  | It costs 9 dollars | No | I’m full |
|  | Please be quiet | Show respect for someone | Sorry |
|  | It costs 7 dollars | Hmm, I don’t think so | It costs 6 dollars |
| Culturally Unfamiliar Gesture | I’ll keep this a secret | Hurry up | It costs 8 dollars |
|  | It’s spicy | Thank you | Are you talking to me? |
|  | I don’t know | Good luck | Okay |
|  | I’m angry | I’m in trouble | It costs 10 dollars |
|  | I’m full | It costs 9 dollars | No |
|  | Sorry | Please be quiet | Show respect for someone |
|  | It costs 6 dollars | It costs dollars | Hmm, I don’t think so |
| Speech Alone | It costs 8 dollars | I’ll keep this a secret | Hurry up |
|  | Are you talking to me? | It’s spicy | Thank you |
|  | Okay | I don’t know | Good luck |
|  | It costs 10 dollars | I’m angry | I’m in trouble |
|  | No (I don’t want) | I’m full | It costs 9 dollars |
|  | Show respect for someone | Sorry | Please be quiet |
|  | Hmm, I don’t think so | It costs 6 dollars | It costs 7 dollars |

Appendix 3. Experiment 2 Japanese Study: Video Content

| Phrase | | | Gestures | |
| --- | --- | --- | --- | --- |
| Meaning | 日本語 | Furigana | Culturally Familiar | Culturally Unfamiliar |
| Yes | はい、そうです。 | hai soudesu | head nod vertically | *Shake head from side to side (Bulgaria)** |
| Come here. | こっちですよ。 | kocchi desu yo | Palm down and move four fingers | *Palm facing self, hand move back and forward (America and China)* |
| Hmm I don’t think so. | うーん、どうかな。 | Umm doo ka naa | Tilt one's head with arms crossed at chest | *Point to self and then cross hands with index fingers up in motion (Nigeria)* |
| Money | お金かかるよ。 | okane kakaru yo | Make a circle with thumb and index finger | *Thumb and index finger pointed up (Mexico)* |
| No thank you | もう結構です。 | moo kekkoo desu | Both palms facing the other person and waiving left/right | *X with arms in front of body and hands in fists* |
| What are you saying? | え、何て言った？ | e, nan te itta? | palm behind the ear | *Closed hand w/ fingers touching, shake back and forth (Argentina)* |
| Stupid | ほんと馬鹿だよね。 | honto baka da yo ne | Index finger circling at side of head | *Index finger pointing at forehead (Netherlands)* |
| Calm down; it’s okay | まあまあ、そんなに怒らないで。 | maa maa sonnani okoranaide | Palms down and move hands up and down | *Index finger and thumb together (sign for money in Japan)* |
| There are 10 people | 十人います。 | juunin imasu | Both hands up; palms up | *Fist closed or t with both index fingers (China)* |
| Bye | じゃあ、また後でね。 | jaa mata ato de ne | Waving hand left and right | *Closing and opening hand (Malaysia)... wave both hand simultaneously (Nigeria)* |
| Want to eat? | 食べに行かない？ | Tabeni ikani? | Use an imaginary chopstick to bring food to your mouth | *Mimic the munching mouth with your fingers, at side of head (Brazil)* |
| I’m in trouble | あ、まずかったなあ。 | aa mazukatta na | Scratching back of head | *Hands laced behind head (China)* |
| Is that a dream? | これ、夢かな？ | kore, yume kana? | Pinch one cheek | *Pinch arm (USA)* |
| Thank you | ありがとうございました。 | arigatoo gozai mashita | Bow forward | *Hand patting heart (Egypt)* |
| My condolences | ご愁傷さまです。 | goshuushoo sama desu | Both palms touching in front of the nose | *Make a cross gesture in front of chest (USA)* |
| Submit it to the teacher | 先生に渡してください。 | sensee ni watasite kudasai | both hands pretending to hold and submit a paper | *One hand pretending to hold and submit a paper (USA)* |
| Go for a drink? | 飲みに行かない？ | nomi ni ikanai? | Fist with the thumb and index finger holding a tiny cup | *Flick hand below the neck (Russia)* |
| That’s a relief | ああ、よかった。 | aa yokatta | Hands crossed on chest and lean forward | *Swipe hand over forehead (As if there were sweat) (USA)* |
| Are you talking to me? | 私ですか？ | watashi desu ka? | Index finger to nose | *Hands to chest (USA)* |
| Okay | 大丈夫だよ。 | daijoobu da yo | Make a circle with the arms over head | *Head right to left shoulder (India)* |
| I’m cute, aren’t I? | かわいいでしょう。 | kawaii desho? | Both index fingers pointing to the cheeks | *Palm up and five fingers closing like picking up a small item (Turkey)* |
| Victory | ばんざーい、ばんざーい。 | banzaai banzaai | Both arms and palms up and down | *Index and middle fingers making a V-sign next to the face* |
| Say cheese | 撮って、はいチーズ。 | totte, hai chiizu | V-sign with index and middle fingers | *Index fingers at edge of lips* |
| No good | こっちはダメです。 | kocchi wa dame desu | Fingers straight and make forearms crossed like an X | *A thumb down* |
| It’s spicy (wasabi) | ワサビ、すごく辛い。 | wasabi, suggoku karai | Holding bridge of nose | *Waving hand over tongue (USA)* |
| Are you seeing a girlfriend? | 彼女に会いに行くの？ | kanojo ni aini ikuno? | Pinky up | *Hand left to right on chest (South Africa)* |

* The country in the parenthesis shows where the gesture is often, but not exclusively, used.

Appendix 4. Experiment 2 Japanese Study: Stimuli Distribution by Test Version

|  | **Version A** | **Version B** | **Version C** |
| --- | --- | --- | --- |
| Culturally Familiar Gesture | That’s a relief | Yes | There are 10 people |
|  | Are you talking to me | Come here | Bye |
|  | Okay | Hmm I don’t think so | Want to eat? |
|  | I’m cute aren’t I? | Money | I’m in trouble |
|  | Victory | No thank you | Is that a dream? |
|  | Say cheese | What are you saying? | Thank you |
|  | No good | Stupid | My condolences |
|  | It’s spicy | Calm down, it’s okay | Submit it to the teacher |
|  | Are you seeing a girlfriend? |  | Go for a drink? |
| Culturally Unfamiliar Gesture | There are 10 people | That’s a relief | Yes |
|  | Bye | Are you talking to me | Come here |
|  | Want to eat? | Okay | Hmm I don’t think so |
|  | I’m in trouble | I’m cute aren’t I? | Money |
|  | Is that a dream? | Victory | No thank you |
|  | Thank you | Say cheese | What are you saying? |
|  | My condolences | No good | Stupid |
|  | Submit it to the teacher | It’s spicy | Calm down, it’s okay |
|  | Go for a drink? | Are you seeing a girlfriend? |  |
| Speech Alone | Yes | There are 10 people | That’s a relief |
|  | Come here | Bye | Are you talking to me |
|  | Hmm I don’t think so | Want to eat? | Okay |
|  | Money | I’m in trouble | I’m cute aren’t I? |
|  | No thank you | Is that a dream? | Victory |
|  | What are you saying? | Thank you | Say cheese |
|  | Stupid | My condolences | No good |
|  | Calm down, it’s okay | Submit it to the teacher | It’s spicy |
|  |  | Go for a drink? | Are you seeing a girlfriend? |
